# Case report: Short-long-short mechanism triggering sustained ventricular tachycardia in a patient with a single-chamber ICD but inhibiting antitachycardia therapy

**DOI:** 10.3389/fcvm.2022.984262

**Published:** 2022-08-26

**Authors:** Christiana Schernthaner, Albert Topf, Lukas J. Motloch, Johannes Kraus, Laurenz Hauptmann, Uta C. Hoppe, Bernhard Strohmer

**Affiliations:** Department of Cardiology, Paracelsus Medical University Salzburg, Salzburg, Austria

**Keywords:** ventricular tachycardia, cardiomyopathy, implantable cardioverter defibrillator, antitachycardia therapy, antitachycardia pacing

## Abstract

**Introduction:**

Short-long-short (SLS) sequences are an important cause of ICD pro-arrhythmia and can initiate both polymorphic and monomorphic ventricular tachycardias (VT). Depending on the programming of a single-chamber ICD, the interplay between SLS sequences and combined VT detection criteria can be responsible for withholding adequate anti-tachycardia pacing (ATP) or shock therapy.

**Methods:**

A 78-year-old patient with ICD was admitted to our emergency department after external cardioversion of a long-lasting VT with hemodynamic compromise. The interrogation of the ICD revealed an SLS sequence initiating a monomorphic VT at a rate of 171 bpm (350 ms). The VT discrimination of the implanted single-chamber ICD was based on the onset and stability criteria as the patient had a history of paroxysmal atrial fibrillation. The ICD was programmed that both criteria had to be met for VT detection and initiation of anti-tachycardia therapy.

**Results:**

Due to the SLS sequence in combination with the programmed VT detection interval, the onset threshold was not fulfilled and inhibited adequate therapy. Some relatively slow VT beats following the SLS sequence resulted finally in a considerable delay in the declaration of the episode onset. As a first step, the threshold for VT detection was programmed to 150 instead of 160 bpm. To avoid SLS sequences and pause-dependent ventricular tachyarrhythmias, VVI backup stimulation was increased from 35 to 55 ppm. Besides, a device-specific algorithm called rate smoothing was activated as a potential preventive feature. On the 3-month follow-up, all sustained VT episodes were detected adequately by the reprogrammed device, resulting in appropriate anti-tachycardia pacing. After further refinement and less aggressive programming of rate smoothing, the patient remained free of symptoms and arrhythmias over a follow-up of more than 2.5 years, particularly since progression to permanent atrial fibrillation and pacing at a lower rate of 60 ppm.

**Conclusions:**

SLS sequences may initiate or trigger VT episodes. Misclassification of the true onset may occur in some ICD devices due to specific programming of VT detection criteria. If both criteria “Onset and Stability” have to be fulfilled, ICD therapy is not delivered despite ongoing VT. Anti-bradycardia backup pacing at a very low stimulation rate may facilitate SLS sequences in patients with ICD resembling a potential pro-arrhythmic mechanism. In case of gradual VT onset with some intervals slower than the programmed VT threshold, the detection rate has to be adjusted down to guarantee appropriate identification of the onset.

## Introduction

Abrupt changes in ventricular cycle length, particularly short-long-short (SLS) sequences, have been identified as an important trigger for VT/VF in patients with an implantable cardioverter-defibrillator (ICD) (1). These sequences can precipitate ventricular tachyarrhythmias with a considerable impact on the patient's quality of life and morbidity. According to literature, 8–15% of all ventricular tachycardia (VT) and ventricular fibrillation (VF) episodes in patients with ICD are associated with occurrence of SLS sequences (2).

We present a rare case of a patient with ICD who was not treated with an ICD because of limitations in the detection of the defined VT onset. Instead, the patient had to undergo emergency cardioversion for termination of a long-lasting VT with hemodynamic compromise.

## Case report

A 78-year-old male patient with ICD was admitted to our emergency department after successful external cardioversion of a sustained monomorphic VT at a rate of 180 bpm. The patient was implanted with a single chamber ICD for 15 years (third ICD pulse generator: Origen Mini; Boston Scientific®) as secondary prevention of sustained VT. The underlying heart disease was ischemic cardiomyopathy with a left ventricular ejection fraction of 45%. He had a history of two myocardial infarctions in the past. Therefore, the patient underwent percutaneous coronary interventions with drug-eluting stent implantations in the right coronary and left circumflex arteries. The baseline ECG showed a sinus rhythm with a right bundle-branch-block pattern (QRS duration 146 ms). The patient had a history of paroxysmal atrial fibrillation and was anticoagulated with warfarin.

**Figure 1:**
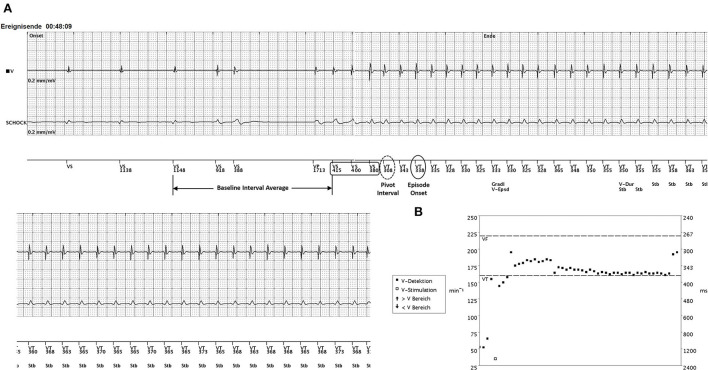
**(A)** The onset of VT after a short-long-short (SLS) sequence. A single ventricular premature beat (coupling interval VS 388 ms) occurs during normal sinus rhythm (52 bpm). The post-extrasystolic pause of 1,713 ms ends with a single ventricular paced beat (VP) at the lower rate limit (35 beats/min) initiating a VT with slight cycle length irregularities at onset (VS 415–380 ms) until a stable cycle length of 350 ms (170 bpm) is present. **(B)** The interval plot of the VT episode with the programmed detection windows (VT threshold 375 ms/VF threshold 273 ms). A sudden onset may be declared “gradual” if the rate transition is five or more beats before the episode onset (third consecutive fast beat = VT 338). Slow “VS” beats (415–400–380 ms) delay the declaration of the episode onset. The pre-onset interval buffer comprises 11 intervals leading to episode onset. The baseline interval average was calculated to be 858 ms (four-interval average beginning with the sixth interval prior to the pivot interval: 918 + 388 + 1,713 + 415 ms = 3,434 ms/4 = 858 ms). The pivot interval is the VT interval at onset with the largest decrease (VS 380–VT 308 ms = 72 ms pivot delta). The second phase interval is determined as the second longest (338 ms) of the four intervals beginning with the pivot interval. The baseline interval average minus the second phase interval was 520 ms. The onset interval was determined as the percentage of the baseline interval average (72/858 = 8%) not exceeding the programmed threshold. Consequently, onset was classified as “gradual” (Gradl V-Epsd), whereas the stability criterion was met. Having the combination onset (9%) AND stability (30 ms) programmed together with SRD OFF, adequate ATP therapy was inhibited until the VT was terminated by external cardioversion after 48 min Strips display the right ventricular electrocardiogram (EGM) on top, the shock EGM below, and the marker channel on the bottom.

During night hours, the patient experienced palpitations with chest pain and dyspnea at rest. The arriving emergency doctor diagnosed a monomorphic VT at a rate of 180 bpm, and external cardioversion had to be performed as the patient presented with hemodynamic deterioration. Even though the clinical VT was faster than the detection rate, the ICD did not deliver any therapies at all. The ICD was programmed at this point of time with two-zone (VT > 160 bpm, 375 ms, Ramp/Scan, 6 × 41 J and VF > 220 bpm, 273 ms, 6 × 41 J) detection using both onset and stability (onset threshold 9%, stability threshold 30 ms) and sustained rate duration (SRD) OFF. The ICD interrogation revealed a monomorphic VT at a CL of 350 ms lasting for 48 min. Interestingly, the arrhythmia was classified correctly by the single-chamber device as stable VT. The VT was preceded by an SLS sequence when a premature ventricular complex (VS coupling interval 388 ms) was followed by a long pause (1,713 ms) ending with a paced ventricular beat (VP, Figure 1). In this type of single-chamber ICD, discrimination can be achieved with onset and/or stability criteria within the given VT detection zone. The ICD was programmed in a way that both criteria had to be fulfilled to initiate therapy. The stability criterion was met according to the stable cycle length (stability 3 ms) and declared the rhythm event as VT. However, the lack of a fulfilled onset criterion prevented the delivery of adequate therapy. Although the episode itself started suddenly, the onset was declared “gradual” because the rate transition was evaluated five or more beats before the episode onset is declared by the device. Slow “VS” beats delayed the declaration of the episode onset that was determined by the third consecutive fast beat “VT 338.” Consequently, VT was diagnosed according to rate and stability; however, the onset percentage of 8% (72/858 ms), calculated by the onset interval (pivot delta 72 ms) as a percentage of the baseline interval average (858 ms), did not exceed the programmed onset threshold of 9%. The pre-onset interval buffer comprises 11 intervals leading to episode onset. The baseline interval average was calculated as four-interval average beginning with the 6th interval prior to the pivot interval (918 + 388 + 1,713 + 415 ms = 3,434 ms/4 = 858 ms). The pivot interval is the VT interval at the onset with the largest decrease (VS 380–VT 308 ms = 72 ms pivot delta). The 2nd phase interval is determined as the second longest (338 ms) of the four intervals beginning with the pivot interval.

The following steps in device reprogramming were taken to prevent the observed phenomenon. The lower rate interval was increased from VVI 35 to 55 ppm to avoid long escape intervals in pacing and potentially arrhythmogenic long-short-long sequences. The rate smoothing feature was set ON 15% below the R-R interval to limit cycle-to-cycle variations; however, one must be aware that rate smoothing is inhibited during hysteresis search cycles and automated sensing tests. Thus, hysteresis correction was set off. The sustained rate duration was programmed ON and set to 5 min as a safety feature. The VT detection zone was lowered from 160 to 150 bpm mainly to improve the onset algorithm performance based on proprietary calculations including the VT rate threshold.

For safety reasons, a coronary angiography was performed to exclude the progression of the existing coronary heart disease and showed a stable result of the coronary two-vessel disease. The anti-arrhythmic drug therapy comprised amiodarone and a beta-blocker and was continued at a sufficient dose. On the 3-month follow-up, device interrogation revealed multiple (67) sustained VTs with adequate detection and delivery of anti-tachycardia pacing therapies (ATPs) preceded by an alleviated SLS mechanism due to rate smoothing. At that time, the underlying heart rhythm of the patient was persistent atrial fibrillation despite the anti-arrhythmic medication.The onset and stability criteria were both fulfilled resulting in appropriate and effective delivery of ATPs. The lower rate limit of bradycardia pacing was further increased to VVI 60 ppm and rate smoothing was set from 15 to 3%. During the 2.5-year follow-up, the patient presented an unremarkable clinical course without any ventricular tachyarrhythmias. Despite the increase in right ventricular pacing, the patient showed no deterioration of his left ventricular function over time.

## Discussion

A short-long-short (SLS) sequence at the onset of VT/VF episodes is a common finding in patients with ICD (1). In the present case, a combination of various peculiarities (SLS mechanism, pacing at a very low ventricular escape rate together with the specific VT detection rate) resulted in a calculated onset percentage that did not exceed the nominal threshold of 9%. As the combination of onset and stability was programmed, therapy was inhibited as the onset was classified as “gradual” despite a stable detected rhythm. If only the stability criterion was programmed, an effective anti-tachycardia treatment would have been delivered as the stability criterion during VT was fully met. However, because the patient presented a history of paroxysmal atrial fibrillation, it was insecure to program the stability criterion only.

The onset is designed to differentiate physiologic from pathologic tachycardias, which typically begin abruptly. It measures the rate of transition in the ventricular rhythm from slow rates to tachycardia. If the rate increase is gradual, it enables the device to inhibit ventricular therapy in the lowest tachycardia rate zone. When a detection window becomes satisfied, the ICD/pulse generator begins calculating for sudden onset in a two-stage sequence. Stage 1 measures the ventricular intervals before the start of the episode and locates the pair of adjacent intervals (pivot point) where the cycle length decreased the most. If the decrease in cycle length is equal to or greater than the programmed onset value, stage 1 will declare a sudden onset. Stage 2 then compares additional intervals. If the difference between the average interval before the pivot point and 3 out of the first 4 intervals following the pivot point is equal to or greater than the programmed onset threshold, stage 2 will declare sudden onset. If both stages declare the rhythm sudden, therapy will be initiated. If either stage indicates a gradual onset, initial ventricular therapy will be inhibited in the lowest zone. Therapy will not be inhibited by onset if the rate accelerates to a higher ventricular zone or if the SRD timer, typically programmed for safety reasons, expires. In a dual-chamber device, therapy will not be inhibited by onset if the atrial lead determines that the RV rate is faster than the atrial rate (V Rate > A Rate programmed ON).

The V Episode Onset algorithm performs the following steps: (1) identify the pre-onset interval buffer (i.e., 11 intervals leading to episode onset treating VP and VS the same), (2) identify the pivot interval from pivot interval candidates, (3) identify baseline interval average and second phase interval, and (4) calculate the onset interval and percentage. The specific algorithm of the device determines the type of VT onset as sudden or gradual and the stability as unstable or stable. If the combination programmed is Onset AND Stability, therapy is inhibited if either parameter indicates that therapy should be withheld. That is, if the rhythm is gradual or unstable the AND condition is not satisfied. However, when these two combinations are used in conjunction with SRD (programmed ON as a safety feature) and the AND conditions are not satisfied, therapy will be inhibited until SRD times out after some minutes.

Notably, a sudden episode may be classified as “gradual” if the rate transition is five or more beats before the episode onset, which is declared by the device as the third consecutive fast beat. The selection of a lower VT rate threshold or adding a VT-1 Monitor Only zone may improve the onset algorithm performance. The onset calculation of the same episode would have resulted in “Sudden” if the VT rate threshold was 140 bpm rather than 160 bpm without any other changes in programming (due to earlier detection of VT onset, 415–400–380 ms) immediately after VP (1,713 ms) increasing the pivot delta to 1,298 ms. In the present case, however, a combination of measures, such as elevation of backup pacing rate (55 ppm), rate smoothing (15%), and lowering of VT rate threshold (150 bpm), prevented the misclassification of VT onset as “gradual”.

The implanted single chamber device has the onset and/or stability criteria for VT discrimination only. An additional morphology criterion might have provided greater sensitivity and specificity for VT detection and treatment (3, 4). Pacing modes that try to avoid right ventricular pacing and backup-pacing at low rates are reported to facilitate SLS sequences (1, 2). Algorithms that avoid unnecessary pacing, as well as capture management algorithms, are known to avoid the risk of right ventricular apical pacing-mediated heart failure, the reason why these algorithms are routinely used nowadays (1). Consequently, individualized programming of bradycardia pacing is crucial to avoid SLS sequences. Algorithms preventing pauses, such as rate smoothing, may reduce VT burden in selected patients (1). However, the Ventricular Arrhythmia Suppression Trial (VAST) failed to show the efficacy of such algorithms in the general ICD population (2). The rate-smoothing algorithm was programmable only in the direction of rate deceleration and was not helpful in reducing the VT burden in the reported case. Antibradycardia pacing may even facilitate SLS sequences because of sudden alterations in ventricular intervals and activation sequences (2). Pacing-induced VT/VF following SLS sequences accounts for 8–15% of all VF/VF episodes in the study by Sweeney et al. In our patient, the rate-smoothing algorithm was initially programmed too high and a pro-arrhythmic effect accounted for many VT episodes. The initiation of VTs was mainly observed after the first paced beat following a decrease of the intrinsic heart rate below the lower rate-pacing limit. This observation may point toward the fact that the patient had a very susceptible substrate in the setting of a ventricular-paced wavefront. Similar cases have been reported after the implantation of a leadless pacemaker (Micra™). In case of a pacing-induced VT catheter ablation of the preexisting myocardial scar should be considered as an effective treatment option (5).

Finally, a reduction in the percentage of rate smoothing together with a moderate increase in lower pacing rate may have contributed to reduction in the VT burden, particularly as the patient developed persistent atrial fibrillation over time. However, one has to keep in mind that suppression of pauses by solely increasing the pacing rate has been reported not to prevent pacing-facilitated VT of VF, and that deactivation of pacing may abolish the phenomenon despite the increased duration of pauses (2, 6).

## Conclusion

The present case highlights the potential pro-arrhythmic effect of SLS sequences occurring before the onset of VT. The SLS phenomenon may interfere with some programmed detection criteria in a single chamber ICD. If the Onset is under-recognized, the device withholds delivery of therapy. Untreated VT may result in serious clinical consequences. Meticulous evaluation of the rate-smoothing algorithm and ventricular anti-bradycardia pacing has to be carried out on an individualized basis. Finally, SRD should always be considered as a bailout feature to guarantee therapy in the VT zone.

## Data availability statement

The original contributions presented in the study are included in the article/supplementary material, further inquiries can be directed to the corresponding author.

## Ethics statement

Ethical review and approval was not required for the study on human participants in accordance with the local legislation and institutional requirements. Written informed consent for participation was not required for this study in accordance with the national legislation and the institutional requirements.

## Author contributions

All authors listed have made a substantial, direct, and intellectual contribution to the work and approved it for publication.

## Conflict of interest

The authors declare that the research was conducted in the absence of any commercial or financial relationships that could be construed as a potential conflict of interest.

## Publisher's note

All claims expressed in this article are solely those of the authors and do not necessarily represent those of their affiliated organizations, or those of the publisher, the editors and the reviewers. Any product that may be evaluated in this article, or claim that may be made by its manufacturer, is not guaranteed or endorsed by the publisher.
